# External validation and logistic recalibration of POSSUM and P-POSSUM for predicting postoperative morbidity and mortality after elective hepatic resection

**DOI:** 10.1186/s12893-026-03508-9

**Published:** 2026-01-26

**Authors:** Niklas Bogovic, Ann-Kathrin Fischer, Miklos Acs, Philipp Kreiner, Hans J. Schlitt, Markus Götz, Stefanie Hofmarksrichter, Paul Kupke, Stefan M. Brunner

**Affiliations:** Department of Surgery, University Hospital Regensburg, Franz-Josef-Strauß-Allee 11, 93053 Regensburg, Germany

**Keywords:** Hepatic resection, Hepatobiliary surgery, POSSUM, P-POSSUM, Risk prediction model, Bootstrap validation, Postoperative morbidity and mortality

## Abstract

**Background:**

Accurate preoperative risk assessment remains critical in hepatobiliary surgery. Established prediction models, such as POSSUM and P-POSSUM, have shown variable performance when applied to specialized procedures. This study externally validated and recalibrated both models to predict postoperative morbidity and mortality after elective hepatic resection.

**Methods:**

All consecutive adult patients who underwent elective hepatic resection at the University Hospital Regensburg between December 2020 and December 2023 were retrospectively analyzed. POSSUM and P-POSSUM scores were calculated using the original logistic equations. Major morbidity (Clavien–Dindo ≥ IIIa) and in-hospital mortality were the predefined outcomes. Model discrimination was assessed using the area under the receiver operating characteristic curve (AUC), and calibration was evaluated using the Brier score, calibration-in-the-large (intercept), calibration slope, and out-of-bag (OOB) calibration plots derived from 1,000 bootstrap resamples. Logistic recalibration was applied to adjust the model intercepts (α) and slopes (β). The clinical utility was evaluated using decision curve analysis.

**Results:**

Of the 200 elective hepatectomies assessed, six were excluded due to missing required physiological inputs, yielding 194 patients with computable predictions. Clinically relevant morbidity (Clavien–Dindo ≥ II) occurred in 146/194 (75.3%) patients, major morbidity (≥ IIIa) in 73/194 (37.6%), and in-hospital mortality in 15/194 (7.7%). Discrimination was fair for morbidity and higher for mortality: AUC 0.696 (95% CI 0.595–0.789) for clinically relevant morbidity, AUC 0.697 (95% CI 0.620–0.764) for major morbidity, and AUC 0.755 (95% CI 0.647–0.851) for in-hospital mortality. OOB bootstrap calibration showed slopes below 1 for all endpoints (clinically relevant morbidity: α 0.16, β 0.837, Brier 0.172; major morbidity: α − 0.051, β 0.907, Brier 0.215; mortality: α − 0.34, β 0.843, Brier 0.068), supporting the need for local model updating.

**Conclusion:**

POSSUM and P-POSSUM can support perioperative risk prediction after hepatic resection when they are locally recalibrated and internally validated. Bootstrap-corrected recalibration yielded stable performance without evidence of overfitting, and decision curve analysis suggested clinical utility across relevant threshold probabilities. These findings support the use of POSSUM-based models in hepatobiliary surgery, provided that centers perform local validation and model updating before implementation in clinical decision-making.

**Supplementary Information:**

The online version contains supplementary material available at 10.1186/s12893-026-03508-9.

## Key points

• The POSSUM and P-POSSUM scores were externally validated in a contemporary cohort of 194 patients who underwent elective hepatic resection at the University Hospital Regensburg and the certified German Liver Center.

• POSSUM showed fair discrimination for morbidity outcomes (AUC 0.697 for major morbidity, Clavien–Dindo ≥ IIIa, and AUC 0.696 for clinically relevant morbidity, ≥ II), whereas P-POSSUM achieved higher discrimination for in-hospital mortality (AUC 0.755).

• Bootstrap out-of-bag validation (1,000 resamples) indicated imperfect calibration with optimism-corrected slopes < 1 (β 0.907, 0.837, and 0.843 for major morbidity, clinically relevant morbidity; β 0.843 and mortality, respectively), supporting the need for local model updating.

• Decision curve analysis suggested a higher net benefit of the recalibrated models compared with “treat-all” and “treat-none” strategies across clinically relevant threshold probabilities, supporting their potential use for perioperative risk communication and institutional benchmarking.

• POSSUM-based risk models can be clinically useful in hepatobiliary surgery, provided that centers perform local validation and recalibration before implementation.

**Supplementary Information:**

The online version contains supplementary material available at 10.1186/s12893-026-03508-9.

## Introduction

Accurate preoperative risk assessment remains a cornerstone of hepatobiliary surgery, where the balance between oncologic radicality and preservation of functional liver parenchyma determines postoperative outcomes [1]. Although advances in perioperative care and the implementation of structured enhanced recovery protocols have improved short-term outcomes, postoperative morbidity after hepatectomy remains clinically relevant in contemporary series, and short-term mortality is typically low in national and multicenter datasets [2, 3]. Reliable preoperative risk estimation is crucial for optimizing patient selection, guiding perioperative management and facilitating informed consent.

Over the past few decades, numerous scoring systems have been developed to quantify surgical risk. Among them, the Physiological and Operative Severity Score for the Enumeration of Mortality and Morbidity (POSSUM) has remained one of the most comprehensive and widely applied tools across surgical disciplines since its introduction in 1991 by Copeland et al. [4]. It combines 12 physiological and six operative parameters to generate estimates of postoperative morbidity and mortality. The Portsmouth modification (P-POSSUM), published in 1996, adjusted the regression coefficients to address overestimation of mortality in lower-risk patients and thereby broadened the model’s clinical applicability [5].

Despite their longevity and broad use, the predictive performances of POSSUM and P-POSSUM have been inconsistent when applied to highly specialized fields such as hepatobiliary and pancreatic surgery. The heterogeneity of liver disease, variable degrees of fibrosis or steatosis, and the extent of parenchymal resection influence both physiological response and postoperative recovery [3, 6, 7]. Previous validation studies have reported that POSSUM may overpredict morbidity in minor resections and underpredict it in extensive or cirrhotic cases [8–10]. A more recent systematic review confirmed that the performance of (P)-POSSUM in major hepatopancreatobiliary surgery is variable and frequently shows limited predictive fit for morbidity, supporting the need for local validation and recalibration [8].

Recently, liver function–oriented risk models have been proposed to improve prediction accuracy beyond generic surgical scores. Among these, indices incorporating biochemical and volumetric parameters—such as the Albumin–Bilirubin (ALBI) grade and the Model for End-Stage Liver Disease (MELD)—have shown a stronger association with postoperative liver dysfunction and mortality following hepatectomy [11]. Similarly, generalized tools, such as the American College of Surgeons National Surgical Quality Improvement Program (ACS-NSQIP) Surgical Risk Calculator, provide institution-independent preoperative risk estimates and facilitate individualized patient counseling [12]. These models improve calibration and risk prediction; however, their need for large datasets and specific biochemical inputs makes them less practical for routine hepatobiliary surgery.

Prediction models such as POSSUM require local validation because their performance depends on the characteristics of the target population and clinical context [13, 14]. With evolving surgical standards and perioperative care pathways, even well-established models may lose their calibration over time. Adjusting the intercept and slope of the logistic equation, a process referred to as recalibration, enables the alignment of predicted probabilities with contemporary data without altering the model’s structure (13,15). Several studies have shown that POSSUM and P-POSSUM often require this type of local adaptation to maintain their validity in hepatopancreatobiliary (HPB) cohorts [6, 8].

In this context, the present study aimed to evaluate the performance of POSSUM and P-POSSUM for predicting postoperative morbidity and mortality after elective hepatic resection in a contemporary single-center cohort, with POSSUM applied to morbidity and P-POSSUM to mortality prediction according to their original design. We hypothesized that POSSUM would retain moderate discriminatory ability and satisfactory calibration for morbidity, whereas P-POSSUM might require recalibration to account for improved perioperative outcomes and lower mortality rates in contemporary hepatobiliary surgery.

## Methods

### Study design and population

This retrospective single-center cohort study included all adult patients who underwent elective hepatic resection at the Department of Visceral Surgery, University Hospital Regensburg, between December 2020 and December 2023. Patients who underwent emergency procedures, combined hepatic and extrahepatic resections, and those with missing required physiological input variables for POSSUM calculation were excluded. All operations were performed by board-certified hepatobiliary surgeons under the supervision of a multidisciplinary tumor board. Perioperative care followed a standardized institutional pathway aligned with Enhanced Recovery After Surgery (ERAS) principles that have been in place since at least 2016 and thus predated the study period; formal adherence to individual ERAS elements was not audited in this retrospective dataset.

### Data collection

Clinical and perioperative data were extracted from electronic medical records and verified using operative reports and discharge summaries. The variables included demographic characteristics, comorbidities, laboratory findings, and operative details, including the extent of resection (major hepatectomy defined as resection of ≥ 3 Couinaud segments; minor hepatectomy < 3 segments). POSSUM and P-POSSUM scores were calculated preoperatively using the original logistic Eqs. (4,5). The predicted morbidity and mortality risks were expressed as continuous probabilities. Postoperative complications were graded according to the Clavien–Dindo classification of surgical complications [15]. For patients with more than one complication, the highest Clavien–Dindo grade during index hospitalization was used for analysis.

The POSSUM and P-POSSUM predicted risks were calculated preoperatively using the original scoring system and logistic equations. The predicted morbidity and mortality risks were expressed as continuous probabilities. To ensure reproducibility, the complete scoring tables and logistic equations used for morbidity and mortality predictions are provided in Supplementary Table S1-S2.

### Handling of missing data

In 6/200 (3%) patients, calculation of POSSUM/P-POSSUM predicted risks was not possible because the required physiological input variables were missing (urea/BUN in 5/6, systolic blood pressure, heart rate, and potassium in 1/6). Therefore, these patients were excluded from the model validation, and analyses were performed as complete-case analyses (*n* = 194).

### Outcome measures

Postoperative complications were assessed during the index hospitalization and graded according to the Clavien-Dindo classification. For patients experiencing more than one complication, the highest Clavien-Dindo grade during the index admission was used for the analysis. The primary outcome was major postoperative morbidity, defined as a Clavien–Dindo grade ≥ IIIa (grades IIIa, IIIb, IVa, IVb, and V). The secondary endpoint was clinically relevant morbidity, defined as Clavien–Dindo grade ≥ II. In-hospital mortality (Clavien–Dindo V) was reported as a separate endpoint and represented a subset of the composite Clavien–Dindo ≥ IIIa outcomes. Reoperations were defined as surgical reinterventions performed in an operating theatre under general anesthesia. Interventional radiology and endoscopic procedures were not considered reoperations, irrespective of anesthesia, and were classified within the Clavien–Dindo system according to the original definition. Secondary outcomes included the duration of intensive care unit (ICU) stay and overall hospital length of stay.

### Ethics approval and consent to participate

This study was approved by the Ethics Committee of the University of Regensburg (reference number 25-4374-104). The committee raised no ethical or legal concerns regarding the conduct of this retrospective study. Given the retrospective design and use of anonymized data extracted exclusively from internal hospital records, the requirement for individual patient consent was waived. This study was conducted in accordance with the Declaration of Helsinki.

### Statistical analysis

Continuous variables are presented as mean ± standard deviation (SD) or median with interquartile range (IQR), and categorical variables are presented as counts and percentages. Model discrimination was quantified using the area under the receiver operating characteristic curve (AUC) with 95% confidence intervals computed using the DeLong method. Calibration was evaluated using the Brier score, calibration-in-the-large (intercept), calibration slope, and graphical calibration based on bootstrap out-of-bag (OOB) predictions across deciles of predicted risk. Logistic recalibration was applied to adjust the intercept (α) and slope (β) of the prediction model using the following equation:

$$\:\mathrm{l}\mathrm{o}\mathrm{g}\mathrm{i}\mathrm{t}\left(\mathrm{o}\mathrm{b}\mathrm{s}\mathrm{e}\mathrm{r}\mathrm{v}\mathrm{e}\mathrm{d}\:\mathrm{o}\mathrm{u}\mathrm{t}\mathrm{c}\mathrm{o}\mathrm{m}\mathrm{e}\right)=\:{\upalpha\:}\:+\:{\upbeta\:}\:\times\:\:\mathrm{l}\mathrm{o}\mathrm{g}\mathrm{i}\mathrm{t}\left(\mathrm{p}\mathrm{r}\mathrm{e}\mathrm{d}\mathrm{i}\mathrm{c}\mathrm{t}\mathrm{e}\mathrm{d}\:\mathrm{p}\mathrm{r}\mathrm{o}\mathrm{b}\mathrm{a}\mathrm{b}\mathrm{i}\mathrm{l}\mathrm{i}\mathrm{t}\mathrm{y}\right)$$

This allows the model to correct for systematic over- or underestimation while maintaining its original structure (13).

Internal validation was conducted using 1,000 bootstrap resamples with out-of-bag (OOB) predictions. Optimism-corrected calibration parameters (α, β) and Brier scores were derived from the bootstrap procedure, and apparent versus corrected estimates were compared to quantify the model stability. Calibration plots display OOB-based observed event rates across the deciles of predicted risk.

For P-POSSUM, calibration was assessed using calibration-in-the-large (intercept), calibration slope, and overall accuracy (Brier score), supported by calibration plots based on bootstrap out-of-bag predictions. Because the number of deaths was limited, the calibration estimates for mortality should be interpreted cautiously. The clinical utility of the recalibrated models was examined using decision curve analysis across threshold probabilities of 0.01 to 0.5 for morbidity and 0.01 to 0.2 for mortality, as described by Vickers and Elkin [16].

### Software

All analyses were conducted using R (version 4.5.0; R Foundation for Statistical Computing, Vienna, Austria) and Python (version 3.13.3; Python Software Foundation, Wilmington, DE, USA) with the pROC, rms, ggplot2, and scikit-learn packages.

## Results

### Cohort description and outcomes

Among the 200 elective hepatectomies performed during the study period, six cases were excluded because the POSSUM/P-POSSUM-predicted risks could not be calculated owing to missing input data, resulting in a final cohort of 194 patients. The median age of the entire cohort was 66 years (IQR, 56–74). A total of 110 patients (57%) were male, and the most frequent ASA class was III, recorded in 116 patients (60%).

The median surgical duration was 251 min (IQR, 190–330). Admission to the intensive care unit (ICU) occurred in 170 patients (85%), with a median ICU stay of 2 days (IQR 2–4). The overall hospital stay was 15 days (IQR, 10–24). In 63 patients (32%), the length of stay exceeded 20 days.

**Fig. 1:**
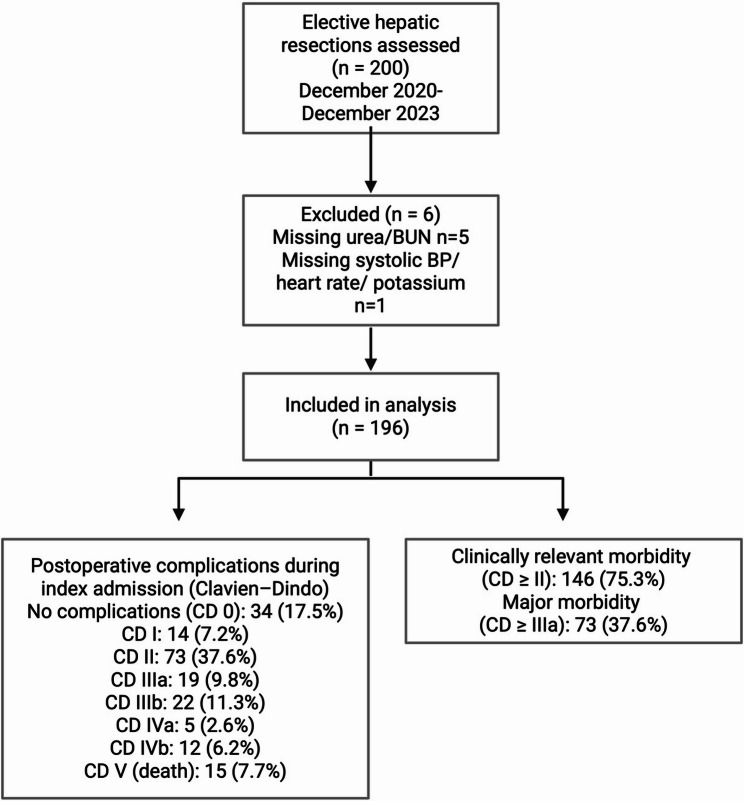
Study flow diagram of the validation cohort. Of the 200 elective hepatic resections assessed, six cases were excluded because the calculation of POSSUM/P-POSSUM-predicted risks was not possible due to missing required input variables (missing urea/BUN, *n* = 5; missing systolic blood pressure/heart rate/potassium, *n* = 1). The final validation cohort comprised 194 patients in total. Postoperative complications during the index admission were graded according to Clavien–Dindo and are shown by grade distribution (CD 0–V). Clinically relevant morbidity was defined as Clavien–Dindo grade ≥ II, major morbidity as Clavien–Dindo grade ≥ IIIa (IIIa–V), and in-hospital mortality as Clavien–Dindo grade V. Graphik created in BioRender. Bogovic, N. (2025) https://BioRender.com/mpzofu0

During the index hospitalization, 37 patients (19%) required reoperation, accounting for a total of 88 procedures (median 2 [IQR 1–3] per reoperated patient). Clinically relevant postoperative complications of Clavien–Dindo grade ≥ II occurred in 146 patients (75%), and major complications (grade ≥ IIIa) were observed in 73 patients (37.6%). In-hospital mortality was recorded in 15 patients (7.7%) (Fig. 1).

A detailed overview of the baseline characteristics, perioperative outcomes, and outcomes during the index admission is presented in Table 1.

**Table 1:** Baseline characteristics of the study cohort (*N* = 194). Data are presented as median [IQR] or n (%). ICU: intensive care unit; LOS: length of stay. The model performance (AUC, calibration, and DCA) was evaluated in a complete-case cohort of 194 patients

Characteristic	Overall (*N* = 194)
Demographics	
Age, years — median [IQR]	66 [56–74]
BMI, kg/m² — median [IQR]	25.6 [23.0–28.1]
Sex, male — n (%)	110 (57)
Sex, female — n (%)	84 (43)
*Perioperative variables*	
ASA I — n (%)	3 (2)
ASA II — n (%)	71 (36.6)
ASA III — n (%)	116 (60)
ASA IV — n (%)	4 (2)
ASA V – n (%)	0 (0)
Extent of resection – n(%)	
Major hepatectomy (≥ 3 segments) — n (%)	126 (64.9)
Minor hepatectomy (< 3 segments) — n (%)	68 (35.1)
Operation duration, min — median [IQR]	251 [190–330]
Surgical reintervention (≥ 1), n (%)	37 (19)
• Total surgical reinterventions — n	88
• Re-operations per re-operated — median [IQR]	2 [1–3]
*ICU and hospital course*	
Any ICU stay — n (%)	169 (87)
ICU length of stay, days — median [IQR]	2 [2–4]
ICU > 2 days — n (%)	90 (46.4%)
Hospital length of stay, days — median [IQR]	15 [11–24]
LOS > 20 days — n (%)	62 (32)
Max ICU / Max hospital stay, days	48 / 90
*Outcomes during index admission*	
No complications Clavien-Dindo 0 – n (%)	34 (17.5)
Clavien-Dindo I — n (%)	14 (7.2)
Clavien-Dindo II — n (%)	73 (37.6)
Clavien-Dindo IIIa — n (%)	19 (9.8)
Clavien-Dindo IIIb — n (%)	22 (11.3)
Clavien-Dindo IVa — n (%)	5 (2.6)
Clavien-Dindo IVb — n (%)	12 (6.2)
Clavien-Dindo V — n (%)	15 (7.7)
Clinically relevant morbidity (≥ CD II) — n (%)	146 (75.3)
Major morbidity (≥ CD IIIa) — n (%)	73 (37.6)
In-hospital mortality — n (%)	15 (7.7)

### Predicted risk (POSSUM and P-POSSUM)

Predicted risks derived from the POSSUM (morbidity) and P-POSSUM (in-hospital mortality) models showed a right-skewed distribution. The median predicted risk of morbidity was 30.8% (IQR 16.9–54.5%), whereas the median predicted mortality risk was 5.6% (IQR 3.0–11.6%).

In the analyzed cohort of 194 patients, postoperative events were common. Clinically relevant postoperative complications of Clavien–Dindo grade ≥ II occurred in 146/194 patients (75.3%). Major morbidity was defined as Clavien–Dindo grade ≥ IIIa, comprising grades IIIa, IIIb, IVa, IVb, and V, and occurred in 73/194 patients (37.6%). In-hospital mortality (Clavien–Dindo V) was recorded in 15/194 cases (7.7%) and represented a subset of the composite Clavien–Dindo ≥ IIIa outcome. The complete Clavien–Dindo distribution was: no complication 34/194 (17.5%), grade I 14/194 (7.2%), grade II 73/194 (37.6%), grade IIIa 19/194 (9.8%), grade IIIb 22/194 (11.3%), grade IVa 5/194 (2.6%), grade IVb 12/194 (6.2%), and grade V 15/194 (7.7%).

These observed rates were higher than the median predicted risks for both endpoints, indicating that the models tended to underestimate the event probability in this population. The subsequent sections present the discrimination, calibration, and decision curve performance of the original and recalibrated models.

### Model discrimination

**Fig. 2:**
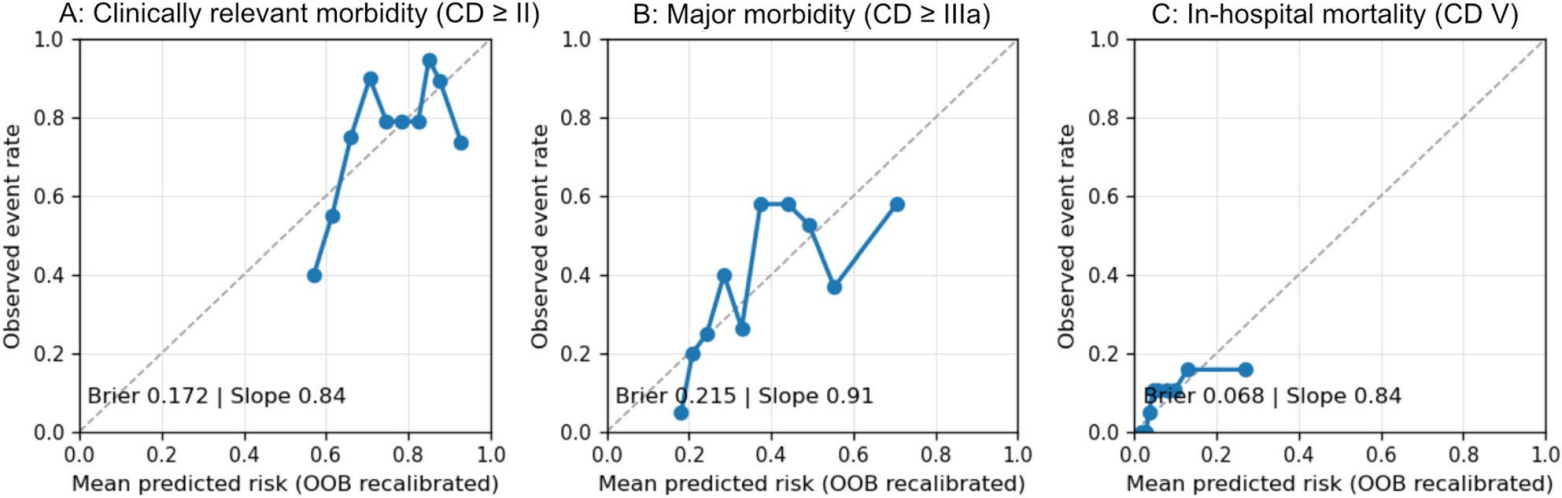
ROC curves for the POSSUM (morbidity) and P-POSSUM (mortality) in patients with computable predictions (*n* = 194). **(A)** Clinically relevant morbidity (≥ CD II), AUC 0.696 (95% CI 0.595–0.789); **(B)** Major morbidity (≥ CD IIIa), AUC 0.697 (95% CI 0.620–0.764); **(C)** In-hospital mortality (CD V), AUC 0.755 (95% CI 0.647–0.851)

Among the 194 patients with computable predictions, the models showed fair discriminatory ability for morbidity and higher accuracy for in-hospital mortality than the other models. For clinically relevant morbidity (Clavien–Dindo ≥ II), 146 events were observed, and the POSSUM morbidity prediction yielded an AUC of 0.696 (95% CI 0.595–0.789). For major morbidity (Clavien–Dindo ≥ IIIa), based on 73 events, the AUC was 0.697 (95% CI: 0.620–0.764). In-hospital mortality occurred in 15 patients, and the P-POSSUM model achieved an AUC of 0.755 (95% CI 0.647–0.851). The corresponding receiver operating characteristic (ROC) curves with 95% confidence intervals are shown in Fig. 2A–C.

As expected, logistic recalibration to improve calibration did not materially alter AUC estimates, as discrimination reflects ranking rather than absolute risk accuracy. Overall, the ROC curves indicated higher sensitivity at lower thresholds for morbidity outcomes with the expected trade-off in specificity, whereas the mortality model maintained a more balanced sensitivity–specificity profile across a wider range of false-positive rates.

Model performance was additionally assessed stratified by the extent of resection (major vs. minor hepatectomy; Table 1). For the prediction of major morbidity (Clavien–Dindo ≥ IIIa) using the POSSUM predicted morbidity risk, discrimination was AUC 0.633 (95% CI 0.535–0.733) in major hepatectomy (*n* = 126, events = 56) and AUC 0.770 (95% CI 0.638–0.885) in minor hepatectomy (*n* = 68, events = 17). Calibration differed between subgroups, with calibration slopes of 0.437(major) and 0.846 (minor) and calibration intercepts of − 0.067 (major) and − 0.456 (minor). Binned calibration plots with bin counts and 95% CIs are provided in Supplementary Figure S1-S2, and detailed subgroup performance metrics are shown in Supplementary Tables S3-S4.

### Calibration and overall accuracy

**Fig. 3:**
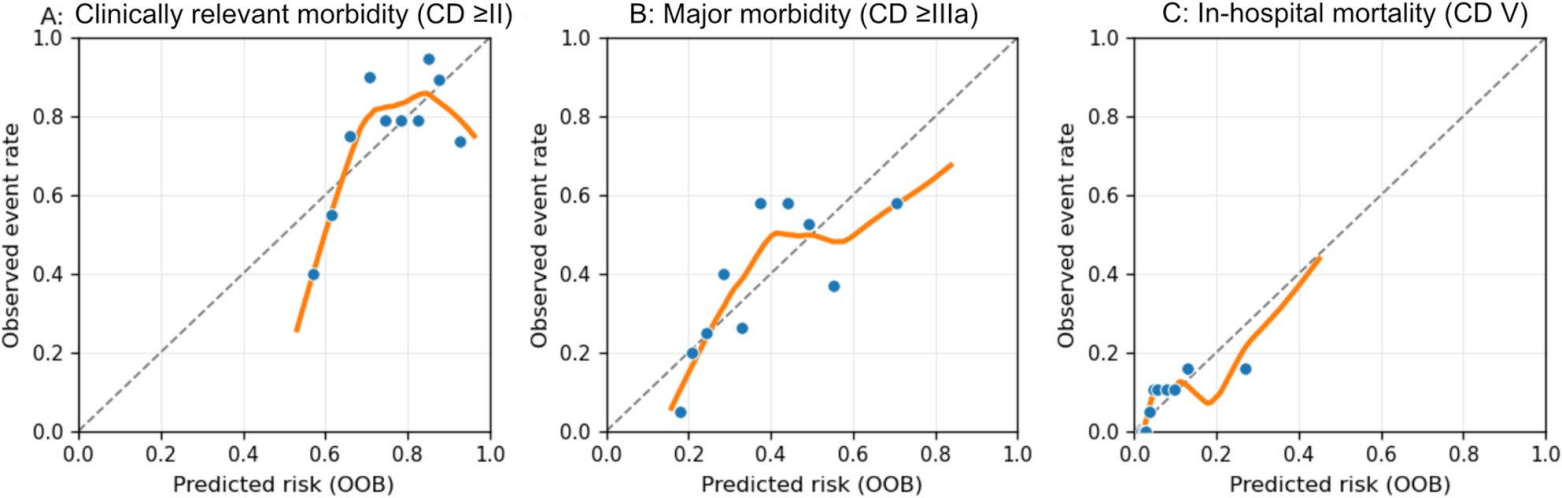
Out-of-bag calibration plots after 1,000 bootstrap resamples for (**A**) clinically relevant morbidity (≥ Clavien–Dindo II), (**B**) major morbidity (≥ Clavien–Dindo IIIa), and (**C**) in-hospital mortality following elective hepatic resection. The dots represent the mean observed event rates per decile of predicted risk; the solid orange lines indicate the loess-smoothed OOB calibration curves; and the dashed diagonal line represents perfect calibration

Calibration was assessed using out-of-bag (OOB) predictions from 1,000 bootstrap re-samples. Figure 3 displays the predicted versus observed event rates for clinically relevant morbidity (≥ CD II), major morbidity (≥ CD IIIa), and in-hospital mortality (Clavien-Dindo V).

For clinically relevant morbidity (≥ CD II), the predicted risks closely matched the observed frequencies across the mid-range. Minor departures appeared only at the distribution tails. Quantitatively, the optimism-corrected calibration intercept was 0.16 (95% CI: 0.112–0.214), and the calibration slope was 0.837 (95% CI: 0.802–0.908), with an optimism-corrected Brier score of 0.172.

Regarding major morbidity (Clavien–Dindo ≥ IIIa), agreement was uniform across most deciles. Slight flattening was visible at higher predicted probabilities, consistent with fewer observations in the upper-risk strata. The optimism-corrected calibration intercept was − 0.051 (95% CI: −0.070 to − 0.016), and the calibration slope was 0.907 (95% CI: 0.885 to 0.948), with an optimism-corrected Brier score of 0.215.

Mortality behaved as expected for a low-event endpoint in this study. The optimism-corrected calibration intercept was − 0.34 (95% CI − 0.445 to − 0.258), and the calibration slope was 0.843 (95% CI 0.828 to 0.900), with an optimism-corrected Brier score of 0.068. Overall, the OOB plots corroborated a stable correspondence between the predicted and observed probabilities within the examined ranges (Fig. 3; Table 2).

**Table 2:** Optimism-corrected calibration parameters from bootstrap out-of-bag (OOB) validation. Calibration intercepts (α) and slopes (β) were optimism-corrected estimates obtained from 1,000 bootstrap resamples using out-of-bag (OOB) validation. Confidence intervals (95% BCa) were derived from bootstrap distributions. The Brier score represents an optimism-corrected measure of the overall prediction accuracy

Endpoint	α_corrected	β_corrected	95% CI (α)	95% CI (β)	Brier score (optimism-corrected)
Clinically relevant morbidity (≥ CD II)	0.16	0.837	0.112 to 0.214	0.802 to 0.908	0.172
Major morbidity (≥ CD IIIa)	-0.051	0.907	−0.070 to − 0.016	0.885 to 0.948	0.215
In-hospital mortality	-0.34	0.843	−0.445 to − 0.258	0.828 to 0.900	0.068

### Clinical usefulness (Decision curve Analysis)

**Fig. 4:**
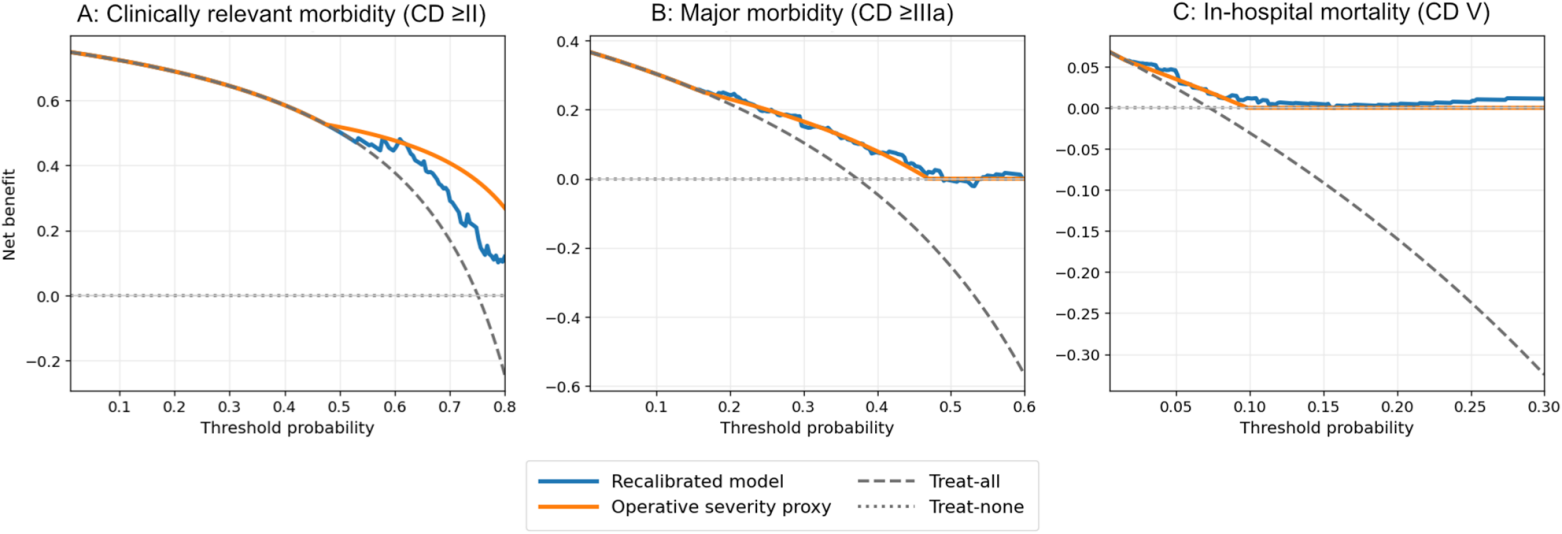
Decision curve analysis for recalibrated models. The panels show the net benefit across threshold probabilities for (**A**) clinically relevant morbidity (Clavien–Dindo ≥ II), (**B**) major morbidity (Clavien–Dindo ≥ IIIa), and (**C**) in-hospital mortality (Clavien–Dindo V). The recalibrated model corresponds to the recalibrated POSSUM for morbidity endpoints (Panels A and B) and recalibrated P-POSSUM for mortality (Panel C). The curves were compared against the treat-all and treat-none reference strategies, as well as an operative severity proxy comparator

Decision curve analysis (Fig. 4) was used to evaluate the net benefit across clinically plausible threshold probabilities and to compare the recalibrated models with treat-all, treat-none, and operative severity proxy models. In Panels 4 A and 4B, the recalibrated model corresponds to the recalibrated POSSUM for morbidity outcomes, whereas in Panel 4 C, it represents the recalibrated P-POSSUM for in-hospital mortality.

For clinically relevant morbidity (Clavien–Dindo ≥ II), within thresholds 0.05–0.50, the recalibrated model showed net benefit values ranging from 0.504 to 0.737. However, the average net benefit was identical to treat-all (mean 0.645 for both strategies), and the recalibrated model did not exceed treat-all or the operative severity proxy at any evaluated threshold within this range (share 0.0% for both comparisons), indicating no incremental benefit beyond these reference approaches for this endpoint (Fig. 4A).

For major morbidity (Clavien–Dindo ≥ IIIa), across thresholds 0.05–0.50, the recalibrated model achieved net benefit values from − 0.006 to 0.339 (mean 0.176). It exceeded treat-all in 76.3% of the thresholds and exceeded treat-none in 97.4% of the thresholds. Compared with the operative severity proxy (mean net benefit 0.173), the recalibrated model was higher in 46.1% of the thresholds, suggesting a modest and threshold-dependent incremental advantage (Fig. 4B).

For in-hospital mortality (Clavien–Dindo V), within thresholds 0.01–0.20, the recalibrated P-POSSUM model yielded net benefit values from 0.00002 to 0.0623 (mean 0.0185). It exceeded treat-all in 97.7% of thresholds (treat-all mean − 0.0403) and exceeded the operative severity proxy in 85.2% of thresholds (proxy mean 0.0147), indicating consistent clinical utility in the low-threshold range relevant to rare but high-impact outcomes (Fig. 4C).

Overall, the decision curves suggest the greatest incremental clinical utility of recalibrated predictions for major morbidity and in-hospital mortality, while for any morbidity, the net benefit was comparable to treat-all and the operative severity proxy within the examined threshold range.

## Discussion

This single-center analysis provides external validation of the POSSUM and P-POSSUM models for predicting postoperative outcomes after elective hepatic resection. In our cohort of 194 patients, POSSUM showed fair discrimination for morbidity (AUC 0.697 for major morbidity and 0.696 for clinically relevant morbidity). Deaths (Clavien–Dindo V) were included in the composite major morbidity endpoint (≥ IIIa) but were reported separately as in-hospital mortality. Calibration assessed by bootstrap out-of-bag predictions demonstrated deviations from perfect agreement, with optimism-corrected calibration slopes below unity (β = 0.907 for major morbidity and β = 0.837 for clinically relevant morbidity) and modest calibration-in-the-large offsets (α = −0.051 and α = 0.16, respectively). The optimism-corrected Brier scores supported adequate overall accuracy. These results suggest that POSSUM remains a useful framework for risk stratification in hepatobiliary surgery, provided that local recalibration is performed prior to use. Similar findings have been reported in other HPB series, where POSSUM demonstrated moderate discrimination and varying calibration depending on case mix and institutional outcomes [7–9, 17].

These findings are clinically relevant because POSSUM is widely used for perioperative risk estimation and institutional benchmarking; however, many centers apply the score without prior local validation. The present study provides such validation specifically for elective liver surgery, a field with limited evidence. Therefore, our results close an important knowledge gap by demonstrating how POSSUM behaves in a contemporary hepatic surgery cohort.

The high observed morbidity rates in this cohort can be explained by the surgical complexity and study period. In the validation cohort, 126/194 (64.9%) procedures were major hepatectomies (≥ 3 segments), a subgroup known to carry a higher complication risk than minor resections. In addition, the study period (2020–2023) overlapped with the COVID-19 pandemic, during which delayed diagnostic pathways were observed in a subset of patients, potentially contributing to more advanced disease at presentation and increased surgical complexity. Pandemic-related delays and pathway disruptions in HPB surgery have been previously described and may have contributed to a more complex case mix during this period [18]. Although several enhanced recovery elements were routinely applied (early mobilization, early oral intake, structured analgesia, and early removal of drains and urinary catheters), a fully standardized ERAS bundle was not implemented throughout the study period. Contemporary ERAS guidelines for liver surgery emphasize bundled implementation; therefore, partial adoption of individual elements may not translate into the morbidity reductions reported in fully standardized ERAS cohorts [19].

Subgroup performance by extent of resection. We evaluated the model performance stratified by the extent of resection, as major and minor hepatectomies represent distinct risk profiles. For the prediction of major morbidity (Clavien–Dindo ≥ IIIa) using POSSUM predicted morbidity, discrimination was AUC 0.633 (95% CI 0.535–0.733) in major hepatectomy (*n* = 126, events = 56) and AUC 0.770 (95% CI 0.638–0.885) in minor hepatectomy (*n* = 68, events = 17). The calibration slopes were 0.437 and 0.846, with intercepts of − 0.067 and − 0.456, respectively, indicating imperfect calibration, with a tendency towards overprediction in the minor subgroup. Given the subgroup sizes and event counts, these findings should be interpreted cautiously. Full metrics and calibration plots are provided in the Supplement.

When the endpoint was broadened to clinically relevant morbidity (Clavien–Dindo ≥ II), discrimination remained comparable (AUC 0.696). Clavien–Dindo grade I events were not analyzed, as they represent minor deviations from the postoperative course without the need for invasive intervention and with limited clinical impact. Calibration showed moderate departures from ideal agreement, particularly at the distributional tails, consistent with the fact that the original model was developed for in-hospital outcomes and was not optimized to capture low-impact deviations from the postoperative course.

In contemporary surgical practice, where enhanced recovery protocols and standardized grading systems, such as the Clavien–Dindo classification, have increased the reporting of minor complications [15, 20], this discrepancy becomes more pronounced. Bootstrap-based calibration analysis yielded similar findings, indicating stable model performance after internal validation, with optimism-corrected intercept and slope estimates consistent with the apparent results. Therefore, our data support the interpretation that POSSUM performs best in predicting major morbidity (≥ CD IIIa), reflecting complications requiring invasive treatment and/or organ support, rather than as a global measure of all postoperative morbidity. This endpoint-specific behavior is consistent with prior HPB work comparing different perioperative scores, where discrimination for severe complications (Clavien–Dindo ≥ IIIa) varied substantially between models [21].

For in-hospital mortality, the P-POSSUM model achieved good discrimination, with an AUC of 0.755. Calibration was not ideal in the bootstrap out-of-bag analysis, with an optimism-corrected intercept of − 0.34 and a slope of 0.843 (Brier score, 0.068), supporting the need for local model updating.

Similar findings have been reported in other HPB and high-volume surgical cohorts. In an elderly HPB cohort, POSSUM tended to overestimate mortality, whereas P-POSSUM provided closer mortality estimates, supporting the concept that score performance depends on case mix and benefits from local updating [22]. Across contemporary surgical series, miscalibration of P-POSSUM has been described, which may reflect temporal and institutional improvements in patient selection, anesthesia, and postoperative critical care relative to the original derivation setting [3, 23, 24].

Our findings support the notion that logistic recalibration can reduce systematic calibration errors without materially changing discrimination [13, 25]. In our cohort, the optimism-corrected calibration parameters for in-hospital mortality were α = −0.34 and β = 0.843, with a Brier score of 0.068, indicating a stable model performance after bootstrap validation.

From a clinical perspective, recalibrated POSSUM estimates may support structured risk communication, guide ICU resource allocation, and facilitate transparent benchmarking in hepatobiliary units. The ability to quantify individualized risk using a well-established and easily applied framework provides a practical advantage, particularly in settings where liver-specific risk calculators are not routinely available. For centers with comparable patient volumes and surgical complexities, model recalibration or integration of liver-specific parameters may further enhance predictive accuracy. Several studies have shown that incorporating markers of hepatic reserve, such as the ALBI grade, MELD score, and residual liver volume, improves outcome prediction after hepatectomy [11, 26]. In addition, preoperative assessment of liver function and resection extent has been demonstrated to correlate closely with postoperative complications and liver failure, underscoring the potential value of hybrid models combining physiological, operative, and biochemical variables [27]. The bootstrap-corrected results provide a useful starting point for further model extensions that integrate liver-specific variables.

Our findings are broadly in keeping with those of a systematic review by Dutton et al. [8], who reported moderate discrimination (AUC 0.65–0.80) and frequent miscalibration of POSSUM and P-POSSUM in HPB surgery. While the present study cannot address external generalizability, it adds quantitative calibration metrics and illustrates that simple intercept and slope adjustments can substantially improve model fit. Model recalibration is widely recognized as a fundamental step in validation and model transportability [13]. These results also provide quantitative evidence of the potential clinical utility. Decision curve analysis suggested that the recalibrated models achieved a higher net benefit than both “treat-all” and “treat-none” strategies across clinically relevant threshold probabilities, supporting their potential value for individualized perioperative risk assessment [16, 28].

This study has several limitations. It was retrospective and single-center in design, and the small number of deaths (*n* = 15) limited the precision of mortality calibration. Six patients were excluded due to missing required POSSUM input variables; given the small number, we did not perform excluded-versus-included comparisons or sensitivity analyses, but complete-case analysis may still have introduced selection bias if missingness was not random. Residual confounding from unmeasured clinical or operative variables cannot be excluded, and we did not distinguish between surgical and medical complications in our study. POSSUM and P-POSSUM also omit contemporary predictors of hepatic reserve and frailty, which may explain residual miscalibration and can be addressed by incorporating liver-specific parameters [11, 26]. Despite bootstrap optimism correction, external validation remains necessary, and our cohort was underpowered for stable subgroup-specific recalibration by extent of resection and temporal validation across 2020–2023.

Taken together, these limitations highlight the need for local validation and model updating before POSSUM-based estimates are used for benchmarking or perioperative risk communication in hepatic surgery.

## Conclusion

In this single-center cohort of 194 elective hepatic resections, POSSUM showed fair discrimination and imperfect calibration for morbidity outcomes, which improved after bootstrap-corrected logistic recalibration, supporting its use for institutional benchmarking and preoperative risk communication when locally validated and updated. Calibration was less favorable when the endpoint was defined as clinically relevant morbidity (Clavien–Dindo ≥ II), highlighting the importance of endpoint-specific validation and recalibration. P-POSSUM achieved good discrimination for in-hospital mortality, and its calibration improved after recalibration. Decision curve analysis suggested potential clinical utility across relevant threshold probabilities. Future multicenter studies incorporating liver-specific predictors (e.g., ALBI, MELD, and volumetric parameters) are warranted.

## Supplementary Information

Supplementary Material 1.

## Data Availability

The dataset supporting the conclusions of this article is not publicly available due to institutional data protection policies but is available from the corresponding author upon reasonable request.
