# Supplementary material for: External validation and logistic recalibration of POSSUM and P-POSSUM for predicting postoperative morbidity and mortality after elective hepatic resection
Source: BMC Surg. 2026 Jan 26;26:80. doi: 10.1186/s12893-026-03508-9 (PMC12849102; doi:10.1186/s12893-026-03508-9)
Supplement: Supplementary file 1 — Supplementary Material 1. [file 12893_2026_3508_MOESM1_ESM.docx]

**Supplementary Material**

**Calculation of POSSUM / P-POSSUM Predicted Morbidity and Mortality Risks**

Risk prediction was based on the Physiological and Operative Severity Score for the enUmeration of Mortality and morbidity (POSSUM) and the Portsmouth modification (P-POSSUM). Each physiological and operative variable is assigned an exponential severity score (1, 2, 4, or 8). The physiological score (PS) is calculated as the sum of 12 physiological variables (Table S1). The operative severity score (OS) is calculated as the sum of 6 operative variables (Table S2).

Predicted risks were calculated using the original logistic regression equations. For any equation, the predicted probability R is obtained as:

R = 1 / (1 + exp(−L)), where L = ln[R/(1 − R)]

The following equations were used:

POSSUM mortality: L = -7.04 + (0.13 x PS) + (0.16 x OS).

P-POSSUM mortality: L = -9.065 + (0.1692 x PS) + (0.155 x OS).

POSSUM morbidity: L = -5.91 + (0.16 x PS) + (0.19 x OS).

Units follow the original score definitions: blood pressure in mm Hg, urea in mmol/L, sodium and potassium in mmol/L, and hemoglobin in g/100 mL (equivalent to g/dL). When multiple descriptors apply within a variable, the most severe category (highest score) was assigned.

**Table S1. POSSUM Physiological Score (12 variables)**

| **Variable** | **Score 1** | **Score 2** | **Score 4** | **Score 8** |
| --- | --- | --- | --- | --- |
| Age, yrs | ≤60 | 61–70 | ≥71 |  |
| Cardiac signs (chest radiograph) | No failure | Diuretic, digoxin, antianginal, or hypertensive therapy | Peripheral edema; warfarin therapy Borderline cardiomegaly | Raised jugular venous pressure Cardiomegaly |
| Respiratory signs (chest radiograph) | No dyspnea | Dyspnea on exertion Mild COPD | Limiting dyspnea Moderate COPD | Dyspnea at rest Fibrosis or consolidation |
| Systolic blood pressure, mm Hg | 110–130 | 131–170 100–109 | ≥170 90–99 | ≤89 |
| Pulse, beats/min | 50–80 | 81–100 40–49 | 101–120 ≥121 | ≤39 |
| Glasgow coma scale | 15 | 12–14 | 9–11 | ≤8 |
| Hemoglobin, g/100 mL | 13–16 | 11.5–12.9 16.1–17.0 | 10.0–11.4 17.1–18.0 | ≤9.9 ≥18.1 |
| White cell count, ×10⁹/L | 4–10 | 10.1–20.0 3.1–4.0 | ≥20.1 | ≤3.0 |
| Urea, mmol/L | ≤7.5 | 7.6–10.0 | 10.1–15.0 | ≥15.1 |
| Sodium, mmol/L | ≥136 | 131–135 | 126–130 | ≤125 |
| Potassium, mmol/L | 3.5–5.0 | 3.2–3.4 5.1–5.3 | 2.9–3.1 5.4–5.9 | ≤2.8 ≥6.0 |
| Electrocardiogram | Normal | Atrial fibrillation (rate 60–90) | Any other abnormal rhythm or ≥5 ectopics/min | Q wave or ST/T wave changes |

**Table S2. POSSUM Operative Severity Score (6 variables)**

| **Variable** | **Score 1** | **Score 2** | **Score 4** | **Score 8** |
| --- | --- | --- | --- | --- |
| Operative severity | Minor | Moderate | Major | Major+ |
| Multiple procedures | 1 | 2 | >2 |  |
| Total blood loss, mL | ≤100 | 101–500 | 501–999 | ≥1000 |
| Peritoneal soiling | None | Minor, serous fluid | Local pus | Free bowel content, pus or blood |
| Presence of malignancy | None | Primary only | Nodal metastasis | Distant metastasis |
| Mode of surgery | Elective | Emergency: resuscitation of >2 hrs possible; operation <24 hrs after admission | Emergency | Immediate surgery <2 hrs needed |

**Table S3. Subgroup model performance by extent of resection (major vs minor hepatectomy).**

| **Outcome** | **Subgroup** | **N** | **Events** | **AUC** | **AUC 95% CI** | **Calibration intercept** | **Calibration slope** | **Brier** |
| --- | --- | --- | --- | --- | --- | --- | --- | --- |
| Major morbidity (CD>=IIIa) | major hepatectomie | 126 | 56 | 0.633 | 0.534–0.725 | -0.067 | 0.437 | 0.251 |
| Major morbidity (CD>=IIIa) | minor hepatectomie | 68 | 17 | 0.77 | 0.633–0.886 | -0.456 | 0.846 | 0.159 |
| Clinically relevant morbidity (CD>=II) | major hepatectomie | 126 | 108 | 0.674 | 0.502–0.828 | 2.075 | 0.484 | 0.342 |
| Clinically relevant morbidity (CD>=II) | minor hepatectomie | 68 | 38 | 0.654 | 0.508–0.783 | 0.671 | 0.417 | 0.319 |
| In-hospital mortality (CD=V) | major hepatectomie | 126 | 13 | 0.675 | 0.544–0.804 | -0.74 | 0.635 | 0.092 |
| In-hospital mortality (CD=V) | minor hepatectomie | 68 | 2 | 0.962 | 0.881–1.000 | 0.842 | 2.414 | 0.02 |

Calibration intercept and slope are based on bootstrap out-of-bag calibration; outcomes refer to events during index admission.

**Table S4. Binned calibration data by subgroup and endpoint.**

| **Subgroup** | **Outcome** | **Bin** | **n** | **Mean predicted risk** | **Observed event rate** | **95% CI low** | **95% CI high** | **Events** |
| --- | --- | --- | --- | --- | --- | --- | --- | --- |
| major hepatectomie | CD>=IIIa | 1 | 13 | 0.123 | 0.077 | 0.014 | 0.333 | 1 |
| major hepatectomie | CD>=IIIa | 2 | 15 | 0.172 | 0.267 | 0.109 | 0.52 | 4 |
| major hepatectomie | CD>=IIIa | 3 | 14 | 0.227 | 0.571 | 0.326 | 0.786 | 8 |
| major hepatectomie | CD>=IIIa | 4 | 11 | 0.297 | 0.364 | 0.152 | 0.646 | 4 |
| major hepatectomie | CD>=IIIa | 5 | 11 | 0.357 | 0.636 | 0.354 | 0.848 | 7 |
| major hepatectomie | CD>=IIIa | 6 | 12 | 0.428 | 0.5 | 0.254 | 0.746 | 6 |
| major hepatectomie | CD>=IIIa | 7 | 13 | 0.524 | 0.462 | 0.232 | 0.709 | 6 |
| major hepatectomie | CD>=IIIa | 8 | 13 | 0.594 | 0.615 | 0.355 | 0.823 | 8 |
| major hepatectomie | CD>=IIIa | 9 | 13 | 0.688 | 0.462 | 0.232 | 0.709 | 6 |
| major hepatectomie | CD>=IIIa | 10 | 11 | 0.873 | 0.545 | 0.28 | 0.787 | 6 |
| minor hepatectomie | CD>=IIIa | 1 | 14 | 0.097 | 0.0 | 0.0 | 0.215 | 0 |
| minor hepatectomie | CD>=IIIa | 2 | 6 | 0.118 | 0.167 | 0.03 | 0.564 | 1 |
| minor hepatectomie | CD>=IIIa | 3 | 6 | 0.134 | 0.333 | 0.097 | 0.7 | 2 |
| minor hepatectomie | CD>=IIIa | 4 | 8 | 0.188 | 0.125 | 0.022 | 0.471 | 1 |
| minor hepatectomie | CD>=IIIa | 5 | 8 | 0.265 | 0.125 | 0.022 | 0.471 | 1 |
| minor hepatectomie | CD>=IIIa | 6 | 11 | 0.383 | 0.364 | 0.152 | 0.646 | 4 |
| minor hepatectomie | CD>=IIIa | 7 | 6 | 0.542 | 0.667 | 0.3 | 0.903 | 4 |
| minor hepatectomie | CD>=IIIa | 8 | 9 | 0.757 | 0.444 | 0.189 | 0.733 | 4 |
| major hepatectomie | CD>=II | 1 | 13 | 0.123 | 0.538 | 0.291 | 0.768 | 7 |
| major hepatectomie | CD>=II | 2 | 15 | 0.172 | 0.733 | 0.48 | 0.891 | 11 |
| major hepatectomie | CD>=II | 3 | 14 | 0.227 | 1.0 | 0.785 | 1.0 | 14 |
| major hepatectomie | CD>=II | 4 | 11 | 0.297 | 0.909 | 0.623 | 0.984 | 10 |
| major hepatectomie | CD>=II | 5 | 11 | 0.357 | 0.909 | 0.623 | 0.984 | 10 |
| major hepatectomie | CD>=II | 6 | 12 | 0.428 | 0.917 | 0.646 | 0.985 | 11 |
| major hepatectomie | CD>=II | 7 | 13 | 0.524 | 0.846 | 0.578 | 0.957 | 11 |
| major hepatectomie | CD>=II | 8 | 13 | 0.594 | 1.0 | 0.772 | 1.0 | 13 |
| major hepatectomie | CD>=II | 9 | 13 | 0.688 | 1.0 | 0.772 | 1.0 | 13 |
| major hepatectomie | CD>=II | 10 | 11 | 0.873 | 0.727 | 0.434 | 0.903 | 8 |
| minor hepatectomie | CD>=II | 1 | 14 | 0.097 | 0.214 | 0.076 | 0.476 | 3 |
| minor hepatectomie | CD>=II | 2 | 6 | 0.118 | 0.5 | 0.188 | 0.812 | 3 |
| minor hepatectomie | CD>=II | 3 | 6 | 0.134 | 0.667 | 0.3 | 0.903 | 4 |
| minor hepatectomie | CD>=II | 4 | 8 | 0.188 | 0.75 | 0.409 | 0.929 | 6 |
| minor hepatectomie | CD>=II | 5 | 8 | 0.265 | 0.625 | 0.306 | 0.863 | 5 |
| minor hepatectomie | CD>=II | 6 | 11 | 0.383 | 0.636 | 0.354 | 0.848 | 7 |
| minor hepatectomie | CD>=II | 7 | 6 | 0.542 | 0.833 | 0.436 | 0.97 | 5 |
| minor hepatectomie | CD>=II | 8 | 9 | 0.757 | 0.556 | 0.267 | 0.811 | 5 |

Bins were constructed across the range of predicted risks; points in calibration plots correspond to these bin summaries.

**Supplementary Figures**

**Supplementary Figure S1. Calibration plots by subgroup.**

| **A** 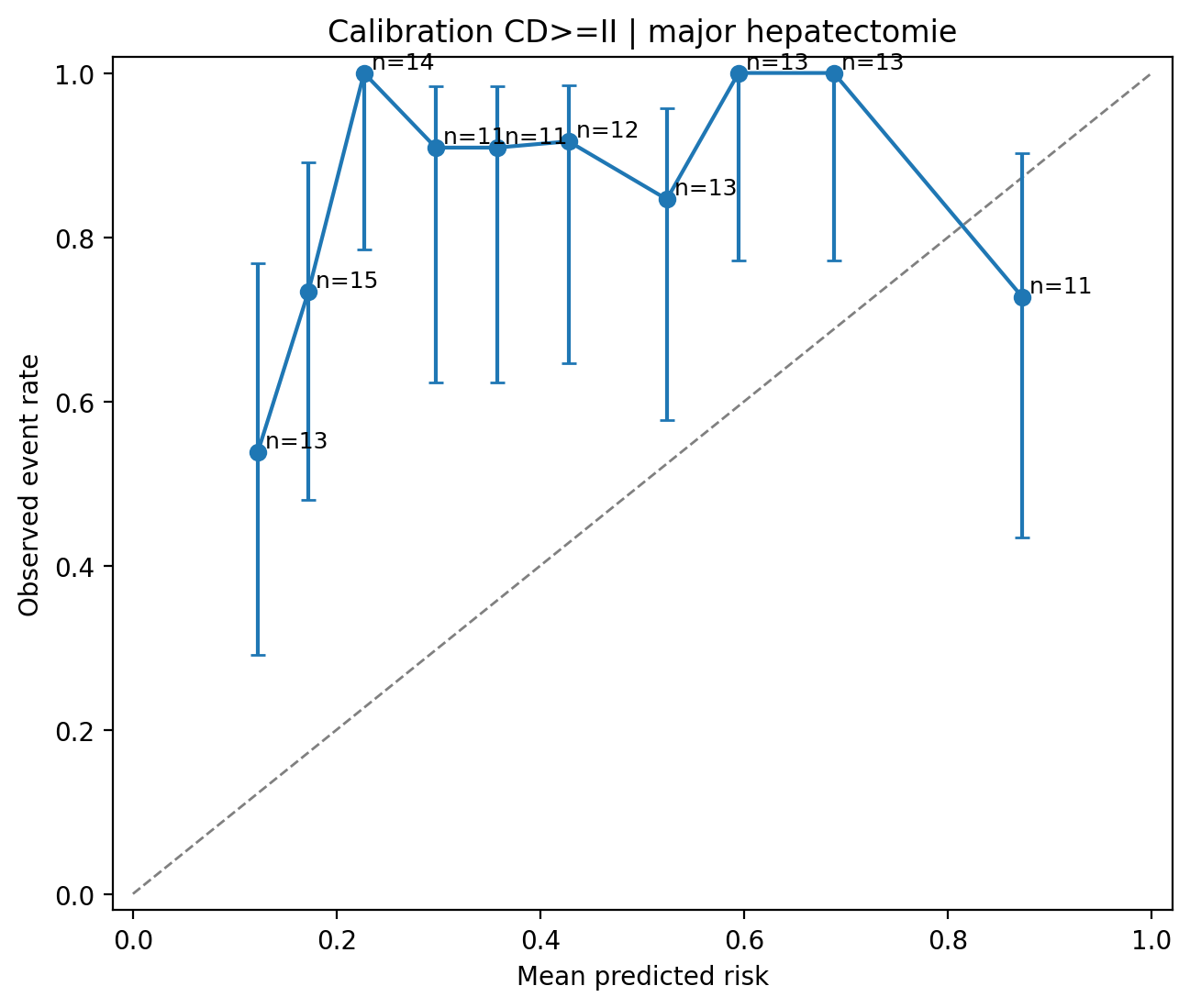 | **B** 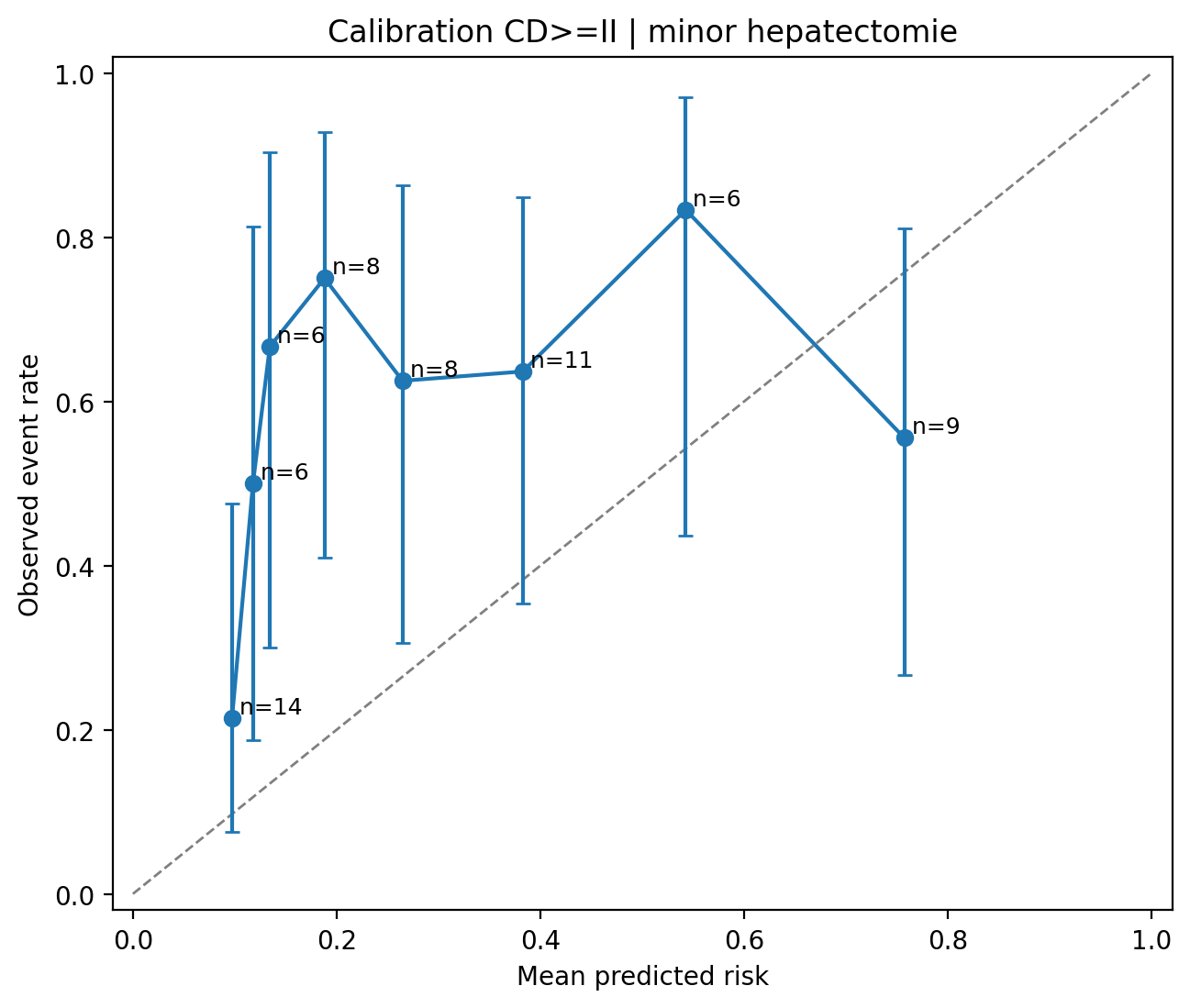 |
| --- | --- |
| **C** 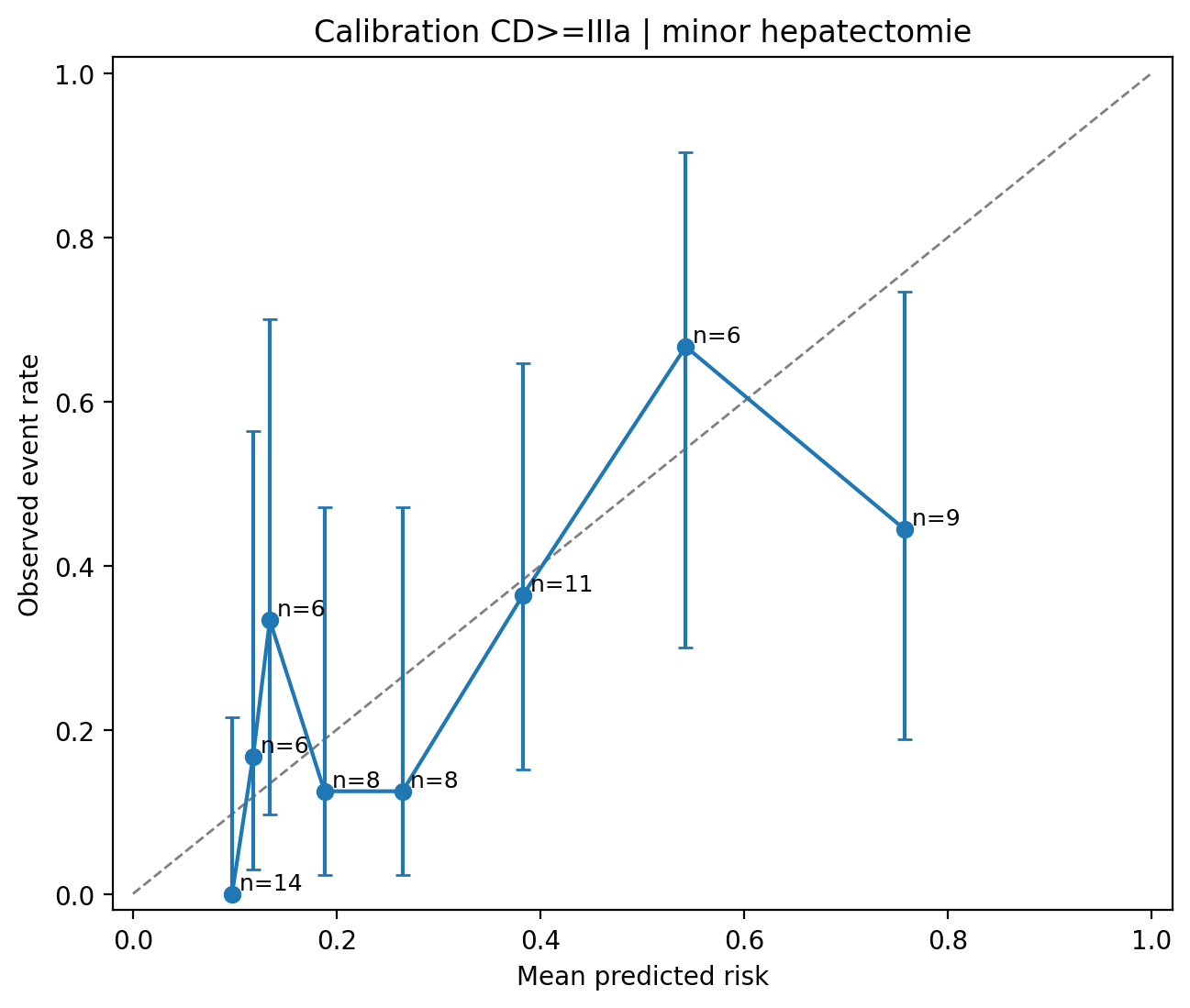 | **D** 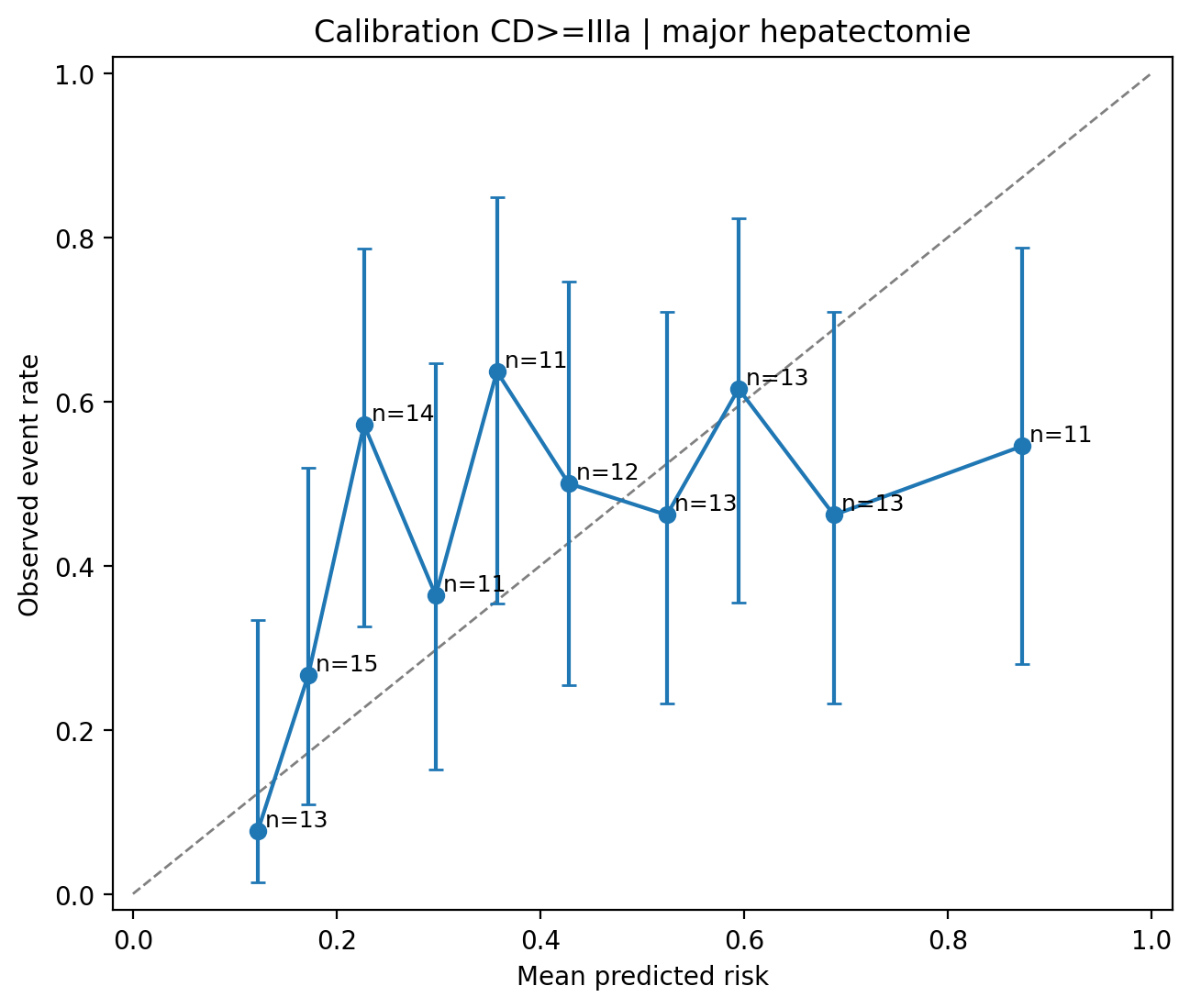 |

Supplementary Figure S1. Binned calibration plots of POSSUM-predicted morbidity by extent of resection. Panels show clinically relevant morbidity (Clavien–Dindo ≥ II) and major morbidity (Clavien–Dindo ≥ IIIa) separately for minor and major hepatectomy subgroups. Points represent mean predicted risk within each bin and the corresponding observed event rate; vertical bars indicate 95% confidence intervals, and labels indicate bin sample size. The dashed line represents perfect calibration.

**Supplementary Figure S2. ROC curves by subgroup.**

| **A** 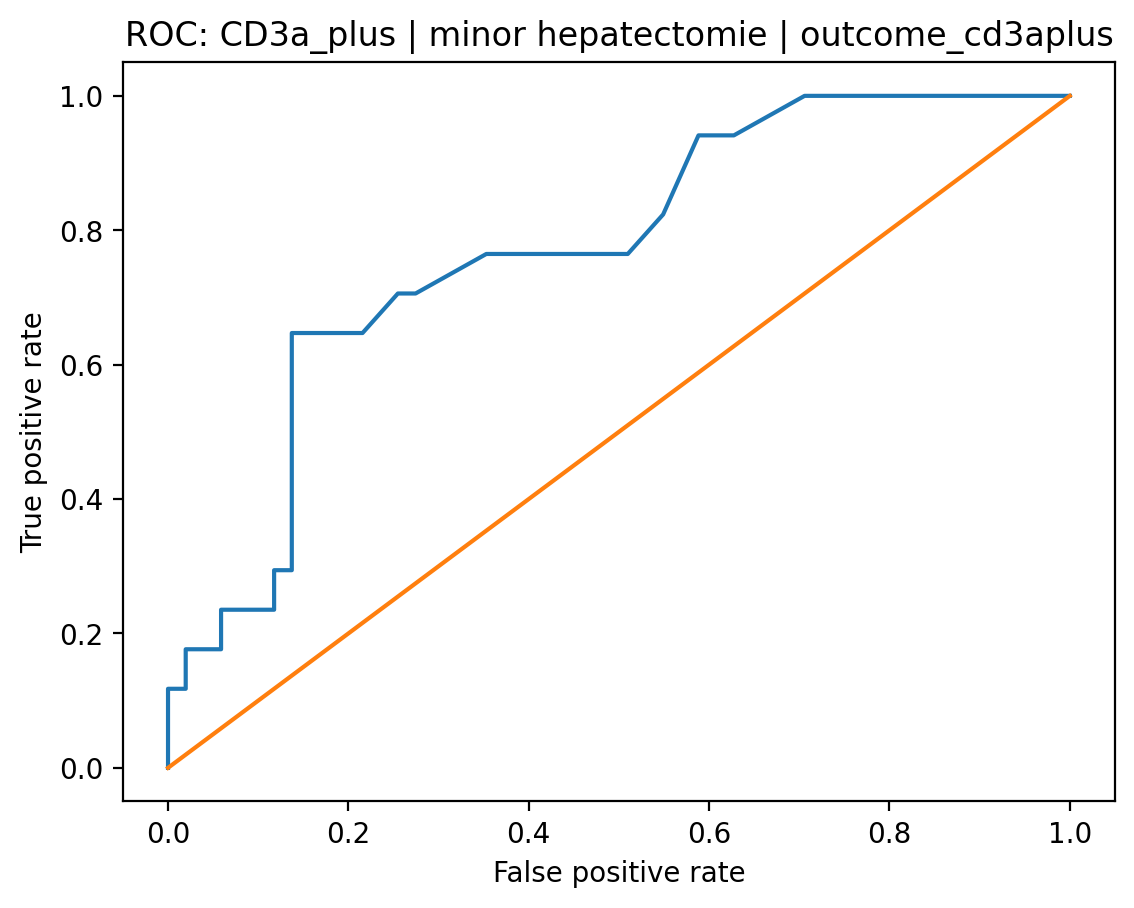 | **B** 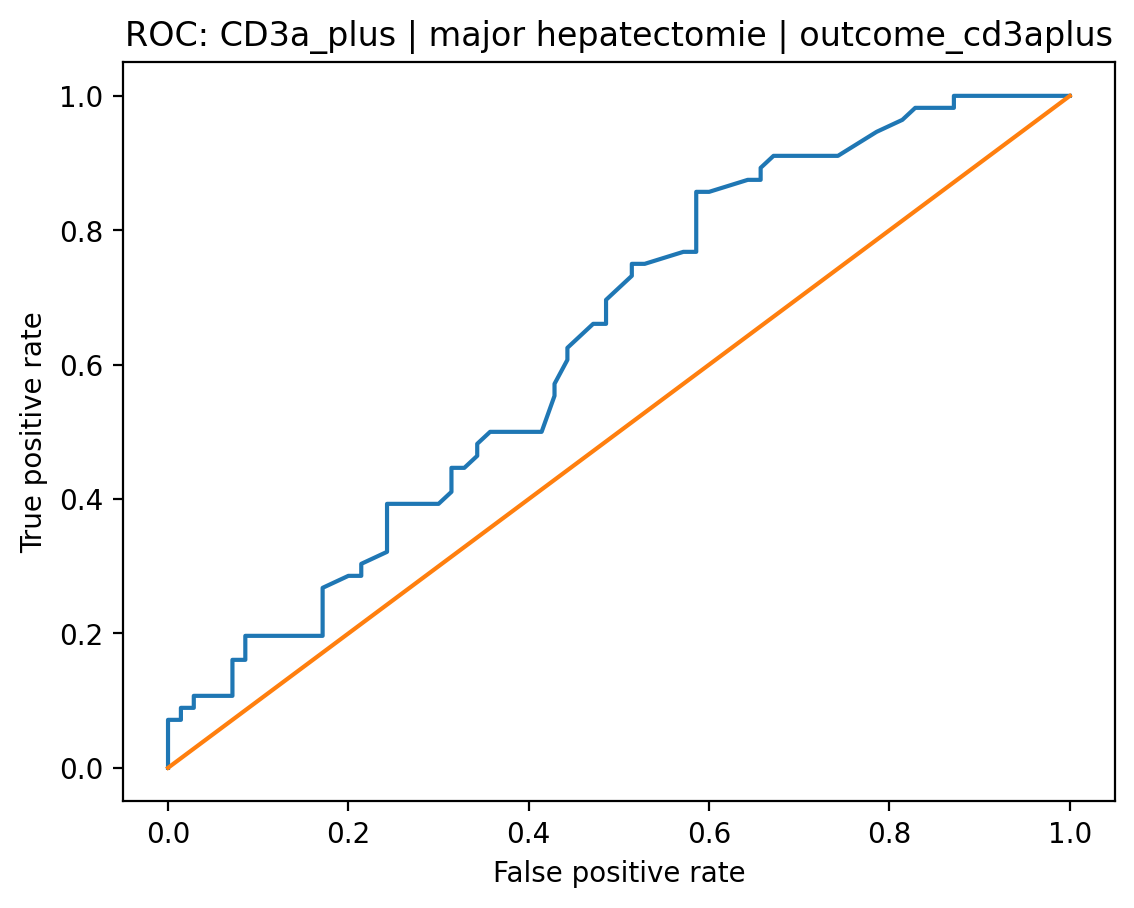 |
| --- | --- |
| **C** 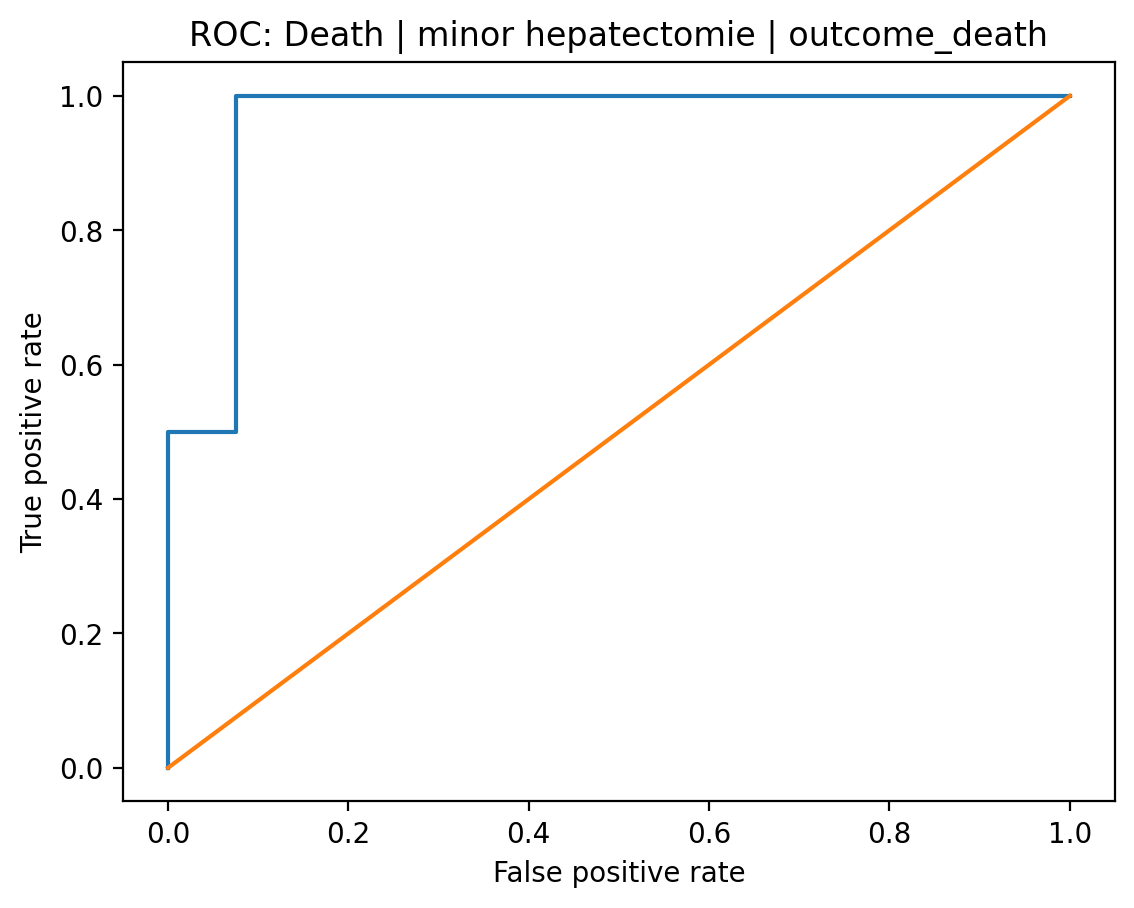 | **D** 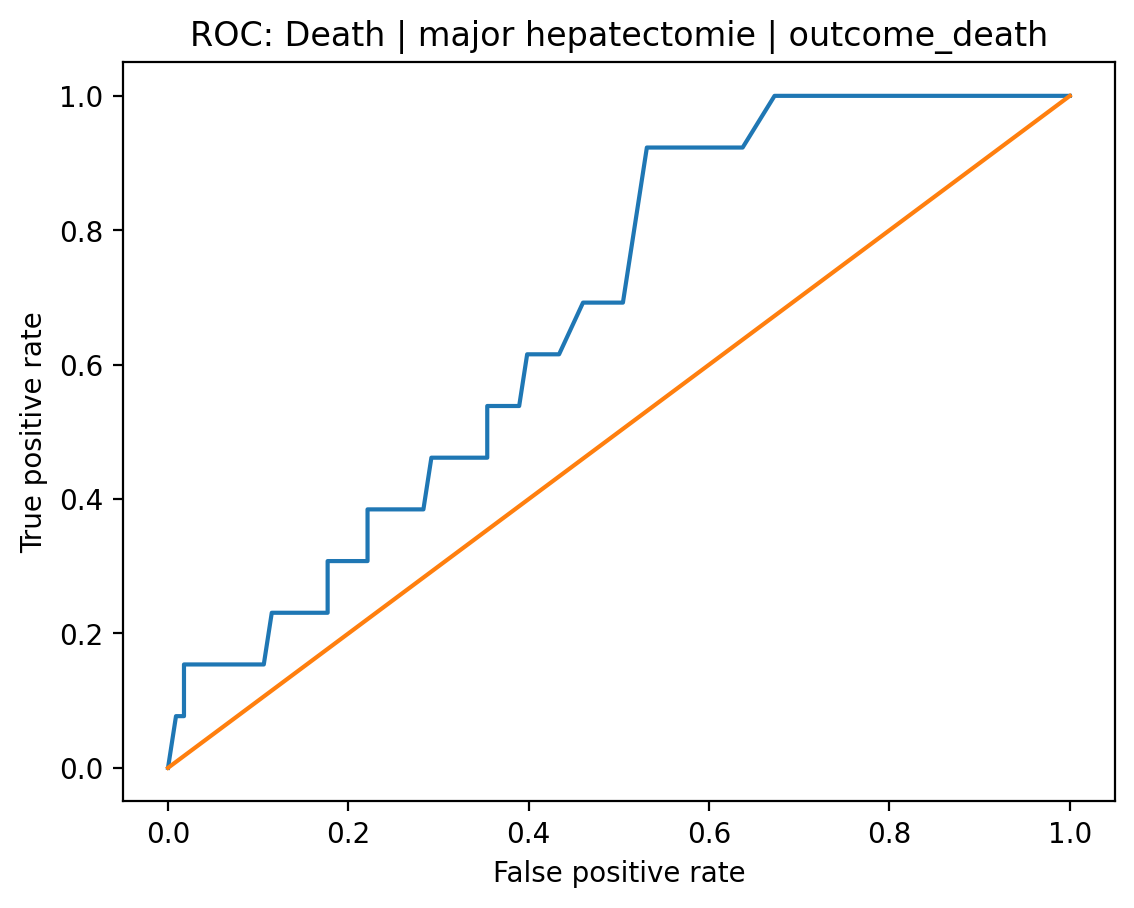 |

Supplementary Figure S2. Receiver operating characteristic (ROC) curves by extent of resection. Panels show discrimination for major morbidity (Clavien–Dindo ≥ IIIa) and in-hospital mortality (Clavien–Dindo V) within minor and major hepatectomy subgroups.
